# Effect of Acute Effort on Isometric Strength and Body Balance: Trained vs. Untrained Paradigm

**DOI:** 10.1371/journal.pone.0155985

**Published:** 2016-05-24

**Authors:** Stanisław Sterkowicz, Janusz Jaworski, Grzegorz Lech, Tomasz Pałka, Katarzyna Sterkowicz-Przybycień, Przemysław Bujas, Paweł Pięta, Zenon Mościński

**Affiliations:** 1 Department of Theory of Sport and Kinesiology, University of Physical Education, Cracow, Poland; 2 Department of Physiology and Biochemistry, University of Physical Education, Cracow, Poland; 3 Department of Gymnastics and Dance, University of Physical Education, Cracow, Poland; 4 Department of Sports Medicine and Human Nutrition, University of Physical Education, Cracow, Poland; 5 Department of Judo, Sport Club GKS Czarni, Bytom, Poland; West Virginia University School of Medicine, UNITED STATES

## Abstract

Years of training in competitive sports leads to human body adaptation to a specific type of exercise. In judo bouts, maintaining hand grip on an opponent’s clothes and postural balance is essential for the effective technical and tactical actions. This study compares changes after maximal anaerobic exercise among judo athletes and untrained subjects regarding 1) maximum isometric handgrip strength (HGSmax) and accuracy at the perceived 50% maximum handgrip force (1/2HGSmax) and 2) the balance of 13 judo athletes at national (n = 8) and international (n = 5) competitive levels and 19 untrained university students. The groups did not differ in age, body height, and weight. Body mass index (BMI) and body composition (JAWON) were evaluated. The Wingate Anaerobic Test (WAnT, Monark 875E) measured recommended anaerobic capacity indices. Hand grip strength (Takei dynamometer) and balance (biplate balance platform) were measured before warm-up (T1), before the WAnT test (T2), and after (T3). Parametric or non-parametric tests were performed after verifying the variable distribution assumption. Judoists had higher BMI and fat-free mass index (FFMI) than the students. The athletes also showed higher relative total work and relative peak power and lower levels of lactic acid. The difference in judoists between HGSmax at T1 and HGSmax at T3 was statistically significant. Before warm-up (T1), athletes showed higher strength (more divergent from the calculated ½HGSmax value) compared to students. Substantial fatigue after the WAnT test significantly deteriorated the body stability indices, which were significantly better in judo athletes at all time points. The findings suggest specific body adaptations in judoists, especially for body composition, anaerobic energy system efficiency, and postural balance. These characteristics could be trained for specifically by judo athletes to meet the time-motion and anaerobic demands of contemporary bouts.

## Introduction

Adequate motor fitness determines the ability to perform self-maintenance activities, professional work, and tourism and recreation activities. Development of motor fitness is particularly essential in professional sport, since its individual aspects or their combinations determine the skill level of athletes. Measuring motor fitness can be useful for strength, speed, and endurance sports. Different situations are observed in sports where motor coordination is more important, such as combat sports, where technical and tactical actions are often performed by athletes under conditions of extreme fatigue (creating disturbances in homeostasis). The fatigue accumulates over subsequent bouts and through incomplete recovery of energy substrates after several bouts.

Judo is a combat sport where throws in standing positions and groundwork fighting are allowed [1]. The duration of a bout for adult judoists is 5 minutes. This is considered as an effective time without breaks. With breaks included, the bout time is longer, with the mean duration reaching 8 minutes. If the bout does not end with an advantage of one of the contestants, extra time is added without any time limit. In elite-level championships, medal winners usually have to take part in 4 to 6 bouts.

This necessitates that the motor fitness of athletes be tested under conditions of extreme fatigue. Some of the tests recommended for examinations of training adaptations are the Wingate Anaerobic Test (WAnT) [2–6], the maximal handgrip strength test [5, 7] and static or dynamic balance test [8–11]. These tests more or less reflect the specific nature of typical phases of a judo bout, and their characteristics have been identified through time motion analysis [12]. Test results have usually been compared for age categories of athletes, [4, 12], competitive levels [2], and, less often, between trained and untrained subjects [10, 13, 14].

The grip is used to transfer forces caused by actions performed during attack or defense to an opponent's body. The specific technical and tactical actions in a judo bout cause the athlete to adapt individual *kumikata* (hand grip on the clothes). After repetition of movements with varied external resistance, the individual differentiation of an athlete’s special motor preparation is achieved [15]. *Kumite* does not occur in the approach phase of a bout (immediately after the *hajime* command) [12], whereas in the gripping phase, the grip force does not necessarily have to be maximal [16]. The gripping phase for adult judoists comprises about half of combat time [12]. Therefore, from the standpoint of technical and tactical actions, grip seems to be essential for success in a bout.

The relationship between the structure and function of grip is reflected by the fact that maximal handgrip strength (HGSmax) has been demonstrated to be positively correlated with body mass [17] and forearm circumference [16]. Seven weight categories are used during competition, and studies have presented HGSmax results for different body mass [2]. Previous findings have demonstrated that HGSmax depends on the age of male judoists, increasing from the cadet category (n = 17, 39.8 ± 12.7 kilogram-force [kgf]) through junior (n = 9, 52.0 ± 8.3 kgf) and adult athletes (n = 17, 57.7 ± 9.0 kgf) [18]. Attempts to interpret this phenomenon by comparing masters and novices have shown that both groups presented different HGSmax. Elite athletes (n = 26) had greater HGSmax values for the right hand (n = 66, 51.0 ± 10.0 kgf vs 42.0 ± 11.0 kgf) and the left hand (49±10 kgf vs 40±kgf) compared to non-elite athletes [2]. Strength endurance has also been shown as an essential factor. Maximal isometric time on *judogi* grip (s) did not depend on sports skill level, whereas a dynamic *judogi* strength endurance test could correctly discriminate between judo athletes from different competitive levels [19].

Gripping is crucial as a means of controlling an opponent’s focus of attention, posture, and balance [20]. Undoubtedly, maintaining balance in a standing position is a determinant for performing strong movement, with effectiveness being improved by intersegmentary coordination during quick changes in situations during a judo bout. The command for starting or resuming a bout (*hajime*) is followed by the approach phase, but closing the kinematic chain with *kumikata* (the gripping phase) is essential for choosing a direction and the manner of performing an individual attack, combination of throws, defense, or counterattack [12].

Judo techniques can be performed while standing on both legs or on one leg. Several studies on this sport have examined the importance of balance using a variety of research models and paradigms. Comparative research strategies are typical in the literature. Analysis of age categories found the best results in static balance in adult athletes, followed by juniors and cadets [11]. In another study, better postural stability was also found in adults compared to junior *judokas* [21]. Postural balance in young *judokas* (12 years old) was significantly better than in their untrained peers [22], which can be interpreted as a synergistic effect of practising this sport. In another study, balance among national and international competitive-level athletes was similar to those at the regional competitive level [8]. Postural balance of elite judoists was superior to controls [23]. Balance examinations have been performed under various conditions (eyes open, eyes closed) [9], and the results have been compared to those of untrained subjects [10, 23].

Judo training should be more specific to the sport since it is characterized by repeated exercise where a high level of anaerobic capacity is needed. Therefore, the WAnT test [2–4, 6, 24] and intermittent exercise for the Special Judo Fitness Test have often been used [6, 24, 25]. Few studies have examined the problem of changes in HGSmax after a judo bout, with post-exercise blood lactic acid (LA) levels ranging from 14.58 to 18.12 mmol/L^-1^. One finding of these studies is that the dominant hand shows an overall decrease in HGSmax after every four successive bouts [26]. However, there are no similar studies of response to judo-specific exercise with respect to changes in balance. Therefore, the aim of this study is to compare HGSmax, the accuracy of the perceived 50% level of HGSmax, and balance indices among judo athletes and untrained peers after performing the maximal anaerobic exercise. Such information may be useful for judo coaches when making professional decisions. We hypothesized that the effect of fatigue caused by maximal anaerobic exercise impairs the structure of control and muscular mechanisms of body stability control. In groups of athletes from sports based on anaerobic exercise, especially intermittent anaerobic exercise, this effect is lower than in the untrained subjects. A correlation is likely to exist between changes in structural stability (exclusion of visual analyzer) and the increase in instability after the exercise compared to the conditions with full control of receptors.

## Material and Methods

### Subjects

Subjects were informed about the experimental risks and signed an informed consent document prior to the investigation. The study was approved by Regional Medical Chamber in Krakow, Poland granted permission No. 108/KBL/OIL/2014. Thirteen judo athletes and 19 untrained university students with similar age (20.24±1.56 years vs. 20.39±0.55 years) participated in the study. The mean training experience of athletes was 13.8±2.7 years, and the athletes trained from 5 to 12 times with an average of 16.3±5.3 hours per week. All of them have won point-scoring places in national-level tournaments, and five of them were included in the ranking of the International Judo Federation (IJF). The total number of the National Judo Federation ranking points at the time of the study ranged from 11 to 325 points. The athletes had been preparing for another competitive period. The examinations were conducted during a training camp in the morning. Subjects were examined at a minimum of 2 hours after a light meal.

### Anthropometry

**Fig 1:**
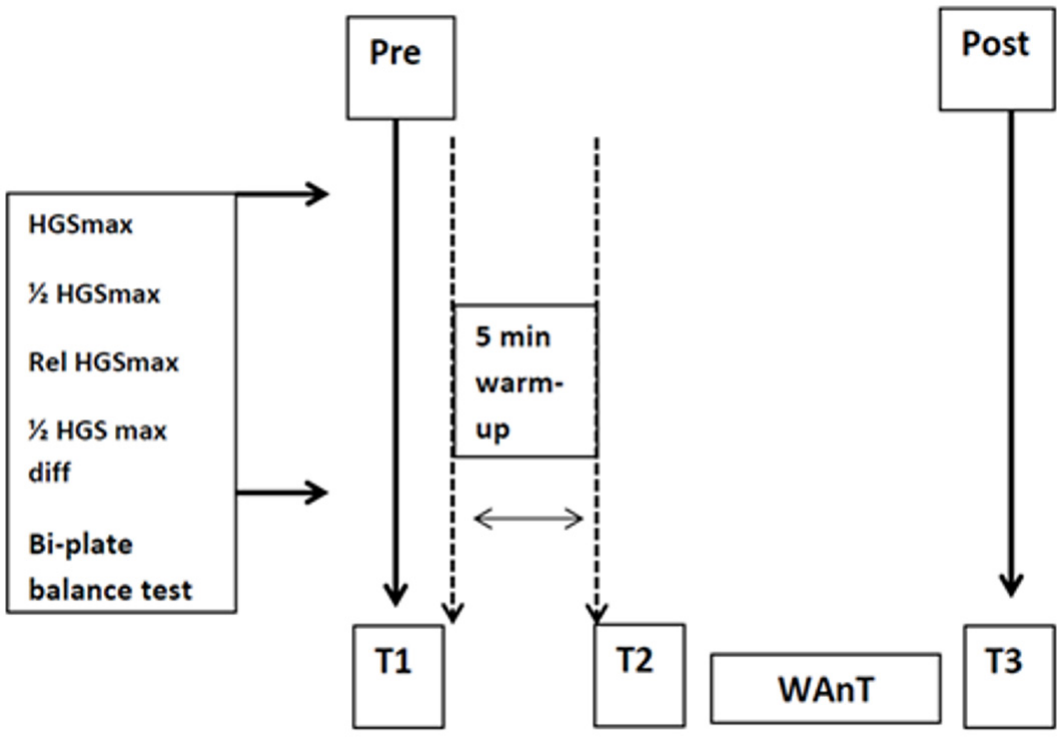
Diagram of tests conducted in the study.

Anthropometric examinations were carried out once. Body height (BH) was measured using a Martin’s anthropometer (USA) with accuracy of 0.5 cm. Body mass (BM), fat percentage (PF%), fat mass (FM in kg), fat-free body mass (FFM in kg), and waist-to-hip ratio (WHR) were calculated using eight-electrode electrical bioimpedance methodology with a body composition analyser (JAWON MEDICAL IOI-353, certificate EC0197, Korea). Measurements of BH and BM were used to compute body mass index BMI (in kg·m^-2^). After computation of fat-free mass index (FFMI) and fat mass index (FMI) (both in kg·m^-2^) [27], we calculated the percentage of fat in the body mass (PF%). Result of measurements are presented in S1 Data. The order of fitness tests performed in the study is illustrated in Fig 1.

### Handgrip strength testing

Handgrip strength (HGS) was measured using a Takei digital grip strength dynamometer (T.K.K. 5401, made in Japan) with an option to adjust the grip to the palm size. The measurements were performed in the standing position with the upper limb kept along the body. At time points T1, T2, and T3 (Fig 1) and after adjustment of the grip, the participant performed one test of HGSmax (in kgf) and a test of the perceived 50% level of maximal grip force (kgf) to examine force sensibility without visual control of the dynamometer's indications. The test was performed with the dominant hand, and all subjects were right-handed. The results were analysed relative to body mass (kgf/kg), and the difference between the obtained and computed force of **½**HGSmax was expressed in kgf and percentages:

$$delta\%=\;\frac{Tdiff}{T/2}\;x\;100$$ (1)

where Tdiff denotes the difference between the obtained and calculated ½HGSmax, and T/2 is the theoretical value of **½**HGSmax at a specific time point (T1, T2, T3).

### Postural balance testing

Postural balance was evaluated using the CQ Stab 2P balance platform (CQ Elektronik System, Poland). The equipment recorded the location of the centre of vertical pressure for forces from 6 sensors (with 3 sensors located in each plate). The sampling rate was 200 Hz per sensor. The platform plates were levelled, and their surfaces were positioned so that they formed one surface. At time points T1, T2, and T3 (Fig 1), 30-second tests were done. The first measurement was for postural stability in a standing position with eyes open (EO). The second test was performed in the same position without visual control of the body position (eyes closed, EC). The width and angle between the legs were natural and not forced in any way. A fixation point was located at a distance of 1 metre opposite the subject. After stepping on the platform, the subject stood still and continued to look at the point of reference. We analysed selected parameters that describe the movement of the centre of foot pressure (COP) (Table 1).

**Table 1:** Parameters used in the study.

Name and Description	Symbol	Metric unit
**(Sway Path) Statokinesiogram path length**	SP	[mm]
**Mean amplitude of COP movement**	MA	[mm]
**Mean velocity of COP movement**	MV	[mm/s]
**Sway area of the COP point**	SA	[mm^2^]
**Mean frequency of COP**	MF	[Hz]

### Wingate anaerobic test (WanT)

For the Wingate anaerobic test [28], a 5-minute warm-up was done beforehand with 5-second accelerations in the 2nd, 4th, and 5th minutes on a Monark 827E cycle ergometer (Sweden). The main part of the test was also performed on the ergometer around 2 minutes after completion of the warm-up. The subjects did a 30-second exercise with a maximum number of rotations with an external resistance of 8.3% of the subject's body mass [29]. During the test, we analysed the relative total work (RTW), relative peak power (RPP), fatigue index (FI), time to obtain peak power (toPP), and time to maintain peak power (tmPP) [28]. Three minutes after the test was completed, fingertip blood samples were taken to determine LA using the enzymatic method with a PAP Lactate test (Lact PAP) by bioMérieux SA (France). The delta LA value was calculated as the difference in measurement values before and after the WAnT test.

Capillary blood samples were taken in both groups in order to evaluate blood plasma L-lactate levels. For this purpose, blood samples were taken from study participants (ca. 80 μl) before the Wingate test and 3 minutes after using capillary tubes and lithium heparin as an anticoagulant on a sterilized fingertip. After blood centrifugation with a rotational speed of 14,000 rpm, 10 μl of blood plasma was sampled from each tube to determine L-lactate levels using the enzymatic method with the PAP Lactate test (Lact PAP) by bioMérieux SA (France). Blood plasma L-lactate levels were expressed in mmol/L.

### Statistics

We performed a formal analysis of power for the available sample size. The examinations in a larger sample of athletes during the competition are almost impossible. Therefore, in our experiment, we performed the measurements among the athletes who were available for participation during the training camp. We did not increase the number of participants in the control group in order not to disturb the proportions. The conventions for small, medium, and large effects were determined following Cohen’s suggestions (30). As Cohen did not specify precisely the conventions for repeated measures, the conventions for between groups effects were applied. The results are presented in the Table 2.

**Table 2:** Power achieved for three sizes of the effect, for between factors and repeated measures.

	Effect size		
	Small (η^2^ = 0.02)	Medium (η^2^ = 0.13)	Large (η^2^ = 0.26)
**Between groups effect**	12%	56%	90%
**Repeated measures effect**	10%	46%	84%

It can be concluded that the power was very good in the case of large effects, less satisfactory in the case of medium sized effects, and unsatisfactory for small effects. Therefore, insignificant results in this study should be interpreted with caution, as some effects may still exist, although very weak. Before comparison of the mean intergroup differences in measurements (judoists; untrained university students), the Shapiro-Wilk test and Student's t-test were performed and the Cohen's ƞ^2^ effect size was evaluated [30]. If the variables did not meet the conditions of equal standard deviations, the t-test for unequal variances was used. Statgraphics Centurion v. 17.2 software was used for calculating the mean and standard deviation along with the median and interquartile range (IQR) if necessary.

Repeated measurements were subjected to factorial repeated measures ANOVA. The Greenhouse-Geisser correction was used if the Mauchly sphericity test was significant. Effect size was also calculated (generalized eta-squared ƞ^2^G) [31]. The criterion for statistical significance was p<0.05 with Bonferroni correction (0.05 / 3 comparisons). Bonferroni confidence limits were used for pairwise contrasts and comparisons. In the case of non-normal distribution, the Friedman test was used for repeated measurements, and the effect size (Kendall’s W) was calculated. Medians were compared using the non-parametric Mann-Whitney test, and the effect size was calculated as r = z/√N (r: r effect size; z: z value; N: number of observations).

## Results

### Body build and body composition

Table 3 presents a comparison of the mean body build and body composition of judo athletes compared to untrained university students. Comparison of the body build and body composition between judoists (n = 13) and university students (n = 19) revealed statistically significant differences in mean BMI (large effect)and FFMI (large effect).

**Table 3:** Body build and body composition of study participants (mean, SD, t, p, ƞ^2^).

	Judokas	Untrained	t	p	ƞ^2^
**BM (kg)**	83.67±20.38	74.13±8.58	1.595	0.132	0.10
**BH (cm)**	178.91±11.99	179.17±5.46	-0.177	0.862	0.00
**BMI (kg·m**^**-2**^**)**	25.90±3.06*	23.15±2.91	2.570	0.015	0.18
**FFM (kg)**	68.23±16.40	60.57±4.38	1.644	0.124	0.11
**FM (kg)**	15.47±5.36	13.61±5.35	0.964	0.343	0.03
**FFMI (kg·m**^**-2**^**)**	21.10±2.32*	18.88±1.27	3.139	0.006	0.29
**FMI (kg·m**^**-2**^**)**	4.80±1.38	4.27±1.77	0.901	0.373	0.03
**PF (%)**	18.36±4.24	17.87±5.28	0.270	0.781	0.00
**WHR (cm/cm)**	0.78±0.04	0.78±0.06	0.323	0.749	0.00

BM–body mass, BH–body height, BMI–body mass index, FFM–fat-free mass, FM–fat mass, FFMI–fat-free mass index, FMI–fat mass index, PF%—percent fat in BM, WHR–waist-hip ratio.

*: p<0.05 for the differences between groups.

### The anaerobic Wingate test

The results obtained from the Wingate test from judoists and untrained university students are presented in Table 4. Comparison of the mean values of the relative total work RTW and relative peak power RPP revealed a significant advantage of judoists over the untrained university students (large effect). Furthermore, the Delta LA level in judokas after the WAnT test was lower than in the untrained subjects with statistical significance (large effect). No differences were found between mean fatigue index.

**Table 4:** Results of the Wingate test for judokas and untrained university students obtained during the experiment (mean ± SD, t, p, Ƞ^2^).

	Judokas	Untrained	t	p	ƞ^2^
**RTW (J·kg-1)**	301.31±19.20*	**262.95±24.31**	**4.756**	**<0.001**	**0.43**
**RPP (W·kg-1)**	12.73±0.95*	**10.89±1.05**	**5.062**	**<0.001**	**0.46**
**FI (%)**	**43.09±6.61**	**39.8±9.63**	**1.068**	**0.294**	**0.04**
**toPP (s)**	**4.86±1.28**	**5.43±1.79**	**-0.978**	**0.336**	**0.03**
**tmPP (s)**	**3.79±0.95**	**4.23±2.26**	**-0.759**	**0.455**	**0.01**
**Delta LA (mmol·L-1)**	11.31±1.71*	**14.23±2.15**	**-0.486**	**<0.001**	**0.36**

RTW–relative total work, RPP–relative peak power, FI–fatigue index, toPP–time to achieve peak power, tmPP–time of maintaining peak power.

*: P<0.05 for the differences between groups.

### Hand grip strength (HGS)

Table 5 presents the measurement results of maximal hand grip strength and the perceived 50% strength before and after completion of the Wingate test. Maximal handgrip strength did not depend on the group factor (F = 0.11, p = 0.748), whereas a significant effect was observed for conditions of performing the test (F = 4.80, p = 0.012, ƞ^2^_G_ = 0.136). Handgrip strength after the WAnT test was significantly higher than during the measurement before the warm-up (T1). A significant difference between time points T1 and T3 was observed in the group of judokas (according to the Bonferroni multiple comparisons test). For HGSmax relative to body mass, no statistically significant intergroup differences were found (F = 2.75, p = 0.108), whereas the effect of the HGSmax test performance on strength was reduced (F = 4.66, p = 0.013, ƞ^2^_G_ = 0.095).

**Table 5:** Maximal hand grip strength and accuracy of perceived half level of strength changes after the Wingate-test (mean ± SD).

	At rest (T1)	After warm-up (T2)	Post-Wingate test (T3)	Condition contrast
**HGSmax (kgf)**	44.05±7.91^c^	44.70±7.81	46.17±7.24^a^	T3>T1
**Judokas**	44.20±10.21	44.66±10.42	47.59±9.32	
**Untrained**	43.95±6.19	44.73±5.71	45.20±5.48	
**Rel HGSmax (kgf/kg)**	0.57±0.098^c^	0.58±0.105	0.60±0.100^a^	T3>T1
**Judokas**	0.54±0.097	0.546±0.109	0.58±0.093	
**Untrained**	0.60±0.094	0.61±0.095	0.62±0.105	
**½ HGS max diff (kgf)**	4.23±7.11^b^^,^^c^	9.29±5.23^a^	9.67±6.84^a^	T3>T1;T2>T1
**Judokas**	7.69±6.57*	7.67±5.70	9.95±5.57	
**Untrained**	1.85±6.61	10.40±4.71	9.47±7.74	
**½ HGS max diff (%)**	17.10±28.48^b^^,^^c^	41.01±21.39^a^	41.09±27.84^a^	T3>T1;T2>T1
**Judokas**	31.97±23.04*	33.32±22.48	40.34±19.73	
**Untrained**	6.92±27.82	46.27±19.47	41.64±32.78	T2>T1;T3>T1

HGSmax–maximal handgrip strength (kgf–kg force). Rel HGSmax–maximal HGS relative to body weight. ½HGS max diff–half exactitude performance changes.

^a^–significantly different from T1 condition;

^b^–significantly different from T2 condition;

^c^–significantly different from T3 condition.

*: P<0.05 for the differences between groups.

The difference in hand grip performance using ½HGSmax was similar between the groups studied (F = 0.37, p = 0.548). The effect size for the experimental factor was high (F = 15.44, p<0.001, ƞ^2^_G_ = 0.332). The result of the measurement of this index (force sensibility) before the warm-up was significantly lower than after the warm-up and after performance of the WAnT test. Analysis of the interactions of the group x conditions of performance of the ½HGSmax test also revealed a significant correlation (F = 10.10, p<0.001, ƞ^2^_G_ = 0.252). A significantly lower difference was found in the group of university students for the first measurement of ½HGSmax compared to the following measurements (T2 and T3). It was also significant for intergroup comparisons (Bonferroni test) at the same time point (T1). At T2 and T3, the intergroup differences were not significant. Using the percentage index, this fact was also demonstrated with respect to the group factor (F = 0.23, p = 0.638), experimental factor (F = 15.28, p<0.001, ƞ^2^_G_ = 0.333), and their combined effect (F = 9.89, p<0.002, ƞ^2^_G_ = 0.248).

### Balance indices

Table 6 presents the results obtained for the measurement of the indices of balance under experimental conditions.

**Table 6:** Balance indices for judokas and university students with eyes open and eyes closed (Median; IQR interquartile range).

	At rest (T1)	After warm-up (T2)	Post-Wingate test (T3)	Condition contrast (Bonferroni 95% intervals)
**SP-EO [mm]**	222.0; 75.0^c^	231.0; 77.5^c^	329.0; 104.5^a^^,^^b^	T3>T2;T3>T1
**Judokas**	185.0; 49.0*	194.0; 36.0*	304.0; 67.0*	
**Untrained**	238.0; 94.0	264.0; 80.0	366.0; 117.0	
**SP-EC [mm]**	300.0; 117.0^c^	282.0; 122.0^c^	378.0; 128.5^a^^,^^b^	T3>T2;T3>T1
**Judokas**	226.0; 71.0*	228.0; 101.0*	349.0; 104.0*	
**Untrained**	330.0; 101.0	292.0; 115.0	413.0; 216.0	
**MA-OE [mm]**	1.85; 1.4	2.25; 1.6	2.07; 1.6	NS
**Judokas**	1.3; 0.8*	2.0; 1.1	2.0; 1.1*	
**Untrained**	2.2; 1.3	2.6; 1.5	3.2; 1.4	
**MA-CE [mm]**	2.5; 1.85	2.15; 1.3	2.9; 1.65	NS
**Judokas**	1.6; 1.6*	2.0; 1.0	2.7; 1.1	
**Untrained**	2.6; 1.7	2.5; 2.1	2.9; 1.65	
**MV-OE [mm/s]**	7.4; 2.25^c^	7.7; 2.55^c^	10.95; 3.5^a^^,^^b^	T3>T2;T3>T1
**Judokas**	6.2; 1.6*	6.5; 1.2*	10.1; 2.2*	
**Untrained**	7.9; 3.1	8.8; 2.7	12.2; 3.9	
**MV-CE [mm/s]**	10.0; 3.9^c^	9.4; 4.1^c^	12.6; 4.25^a^^,^^b^	T3>T2;T3>T1
**Judokas**	7.5; 2.4*	7.6; 3.4*	11.6; 3.5*	
**Untrained**	11.0 3.4	9.7; 3.8	13.8; 7.2	
**SA-EO [mm**^**-2**^**]**	130.0; 111.5^c^	167.5; 107.0^c^	276.0; 235.0^a^^,^^b^	T3>T2;T3>T1
**Judokas**	87.0; 69.0*	133.0; 69.0*	173.0; 127.0*	
**Untrained**	173.0; 127.0	185.0; 239.0	325.0; 282.0	
**SA-EC [mm^2]**	180.0; 196.5^c^	168.5; 140.5^c^	322.0; 172.5^a^^,^^b^	T3>T2;T3>T1
**Judokas**	104.0; 114.0*	120.0; 59.0*	239.0; 120.0*	
**Untrained**	290.0; 183	209.0; 251.0	344.0; 383.0	
**MF-EO [Hz]**	0.675; 0.36	0.555; 0.35	0.64; 0.285	NS
**Judokas**	0.79; 0.43	0.47; 0.31	0.66; 0.44	
**Untrained**	0.66; 0.33	0.56; 0.35	0.63; 0.20	
**MF-EC [Hz]**	0.655; 0.37	0.665; 0.375	0.745; 0.305	NS
**Judokas**	0.7; 0.64	0.66; 0.5	0.76; 0.37	
**Untrained**	0.62; 0.29	0.67; 0.31	0.73; 0.32	
**RQSP**	0.795; 0.225^c^	0.855; 0.230	0.880; 0.145^a^	T3>T1
**Judokas**	0.850; 0.250	0.840; 0.170	0.870; 0.080	
**Untrained**	0.730; 0.210	0.900; 0.210	0.940; 0.150	

*–statistically significant difference in time point; EO–eyes open, EC–eyes closed, SP–sway path, MA–mean amplitude, MV–mean velocity, SA–sway area, MF–mean frequency, RQSP = SPEO/SPEC,

^a^–significantly different from T1 condition,

^b^–significantly different from T2 condition,

^c^–significantly different from T3 condition.

#### Sway Path (SP)

The effect of the experimental factor was significant for the CoP sway path, which depended on the factor of measurement conditions with statistical significance (Friedman test = 39.42. p<0.001, Kendall’s W = 0.62). The CoP sway path after the Wingate test (T3) was significantly longer than the pre-test values (T1 and T2) (95% Bonferroni intervals). The CoP sway path with eyes open depended on the group factor at the first time point (at rest, z = 3.146, p<0.002, r = 0.56), the second time point (After warm-up, z = 3.146, p<0.002, r = 0.56), and the third time point (Post Wingate test z = 3.319, p<0.001, r = 0.35). The CoP sway path in the groups of judokas was significantly shorter than in university students for each time point.

With eyes closed, the effect of the experimental factor on CoP sway path was significant, but a large effect of the factor of conditions observed for eyes open was reduced (Friedman test = 29.68, p<0.001, Kendall’s W = 0.46). After the WAnT test (T3), the CoP sway path was significantly increased compared to the results for T1 and T2. The length of SP in judokas was significantly shorter than in students. The effect size of the group factor was medium for each time point (z = 2.820, p = 0.025, r = 0.49; z = 2.245, p = 0.026, r = 0.40 and z = 2.341, p = 0.018, r = 0.41, respectively).

#### Mean Amplitude (MA)

A significant dependency on the conditions of the balance test was found for the mean amplitude of CoP with eyes open (Friedman test = 9.772, p = 0.007, Kendall’s W = 0.15). Differences between T1 and T3 were significant (95% Bonferroni intervals). Mean CoP amplitude in the group of judokas was significantly lower than in the group of university students at T1 (z = 2.30, p = 0.033, r = 0.38) and T3 (z = 2.379, p = 0.018, r = 0.42), but not at T2 (z = 1.573, p = 0.115, r = 0.28). The factor of experimental conditions (T1, T2, T3) did not have high effect on the value of mean amplitude of CoP with eyes closed (Friedman test = 6.705, p = 0.035, Kendall’s W = 0.10). MA in judokas was shorter than in the untrained subjects. Intergroup difference was significant at T1 (z = 2.130, p = 0.035, r = 0.38), but not at T2 and T3 (z = 1.820, p = 0.071, r = 0.32 and z = 1.746, p = 0.081, r = 0.39, respectively).

#### Mean Velocity (MV)

The effect of the condition factor on MV was large (Friedman test = 39.79, p<0.001, Kendall’s W = 0.62). Mean MV was significantly higher after the WAnT test than pre-test values. The intergroup differences in MV were statistically significant at T1,T2, and T3 (z = 3.127, p = 0.002, r = 0.55; z = 3.108, p = 0.002, r = 0.55; z = 3.281, p = 0.001, r = 0.58, respectively). A medium effect of the factor of experimental conditions on MV was observed for maintaining the balance with eyes closed (Friedman test = 26.69, p<0.001, Kendall’s W = 0.46). MV was significantly higher at T3 compared to T1 and T2. Group means for the same conditions showed significant differences at T1, T2, and T3 (z = 2.820, p = 0.005, r = 0.50, z = 2.206, p = 0.029, r = 0.39, z = 2.341, p = 0.020, r = 0.41, respectively), with MV values lower in the group of judokas compared to the untrained subjects.

#### Sway Area (SA)

The SA of CoP during the test with eyes open depended significantly on the experimental factor (Friedman test = 29.25,p<0.001, Kendall’s W = 0.46). The difference in SA was significant between T3 and T2 and between T3 and T1. SA was the highest at T3. The mean value for this balance index was significantly lower in judokas compared to university students. Comparison of the group means in the same conditions revealed significant intergroup differences at T1, T2, and T3 (z = 2.609, p = 0.010, r = 0.46, z = 2.552, p = 0.011, r = 0.45, z = 2.705, p = 0.007, r = 0.48).

During the test with eyes closed, the SA index depended on the experimental factor (Friedman test = 14.661, p<0.001, Kendall’s W = 0.23). SA was the highest at T3 and significantly different from T2 and T1 (95% Bonferroni intervals). At T3, mean SA during balance test with eyes closed was significantly lower in the group of judokas than in the control group (z = 2.187, p = 0.030, r = 0.39).

#### Mean Frequency (MF)

The effect of the condition factor on MF in the balance test performed with eyes open was insignificant (Friedman test = 1.712, p = 0.408 > p = 0.017, Kendall’s W = 0.028). Comparisons between the groups for the same condition were insignificant (p>0.05). Furthermore, no significant effects of condition factor (Friedman test = 1.54, p = 0.443, Kendall’s W = 0.024) and group factor (p>0.189) on MF were found during the balance test with eyes closed.

#### Romberg Quotient (RQSP)

ANOVA results showed that the performance condition factor did not have a significant effect on RQSP value (Friedman test = 3.606, p = 0.165, Kendall’s W = 0.10).

#### Romberg MV (RQMV)

RQMV (eyes open / eyes closed) did not differ significantly between individual time points (Friedman test = 7.197, p = 0.027, Kendall’s W = 0.11)

## Discussion

**Fig 2:**
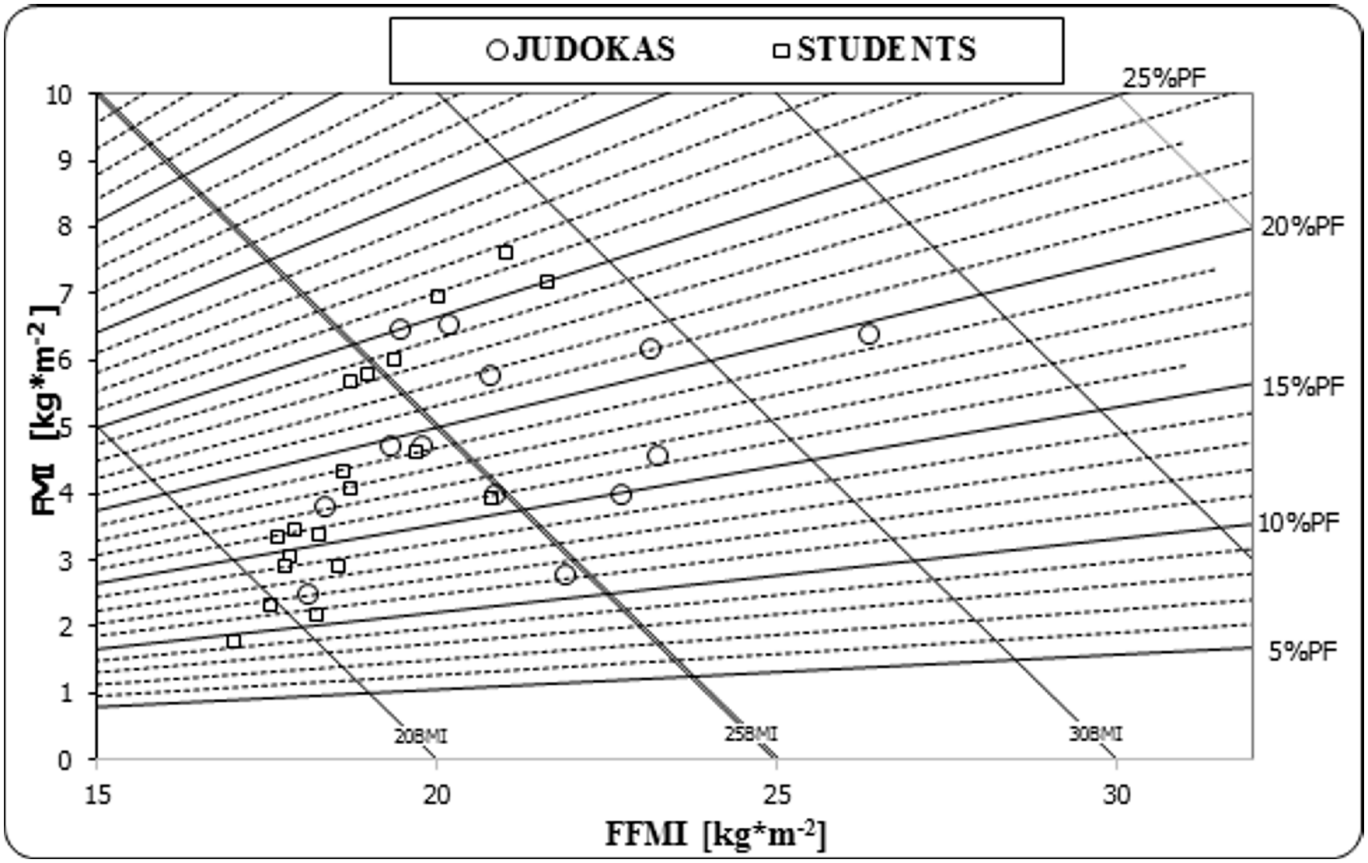
Body composition chart in judoists and untrained university students. BMI–body mass index, FFMI–fat-free mass index, FM–fat mass index, PF%—percent fat in body mass.

BMI and FFMI indices were higher in judoists compared to untrained university students. No significant differences were found between groups for FMI values. Standard values of BMI (24.99 kg·m^-2^) were also exceeded in groups of Brazilian judoists (25.89 kg·m^-2^) and ju-jitsu athletes (26.44 kg·m^-2^) [32]. In our study, these high values were attributable to increased FFMI rather than increased fat percentage (PF%). In the group of untrained subjects, all four dimensions illustrated in the body composition chart (Fig 2) showed high correlations with each other. Waist–hip ratio was very similar between groups (0.78) and within the low-risk category for young healthy men (WHR < 0.83) [33].

Judo athletes had higher values of RTW and RPP and lower LA levels compared to the untrained university students. Generating energy in anaerobic processes during short efforts is determined by phosphocreatine stores, its rate of breakdown, and lactic acid build-up [34]. Elevated lactate concentration leads to disturbances in acid-base balance that might cause disturbances in neuromuscular coordination [35]. Changes in LA concentration are also used to evaluate the contribution of glycogenolysis to produce energy during muscular work. High LA levels show that glycolytic processes play an important role in the energy production process during physical exercise. In practice, exercise economy is essential, and with the same or slightly lower level of homeostasis disturbances, better physical capacity is reflected by higher RTW and RPP [36]. This observation is consistent with the results of our study, where untrained subjects had higher LA levels after the WAnT test and lower RTW and RPP compared to the trained subjects.

In judo-specific physical exercise, the most important role should be attributed to the properties that determine the capacity and power of the glycolytic anaerobic source. Previous studies on the best Polish judokas can be considered as model values for these indices [24, 37–38]. It can be concluded that total work (RTW) and peak power (RPP) were very high in the group of athletes (Table 4).

Anaerobic capacity has been shown to influence most of the stability indices [39], which is consistent with the results in our study. This observation is also reflected by the noticeable differences in RPP, which is an indicator of the phosphagen component of anaerobic capacity, and RTW. In judokas, mean RTW was 301.24 J·kg^-1^ and RPP was 12.73 W·kg^-1^, whereas in the untrained subjects, these values were 262.95 J·kg^-1^ and 10.89 W·kg^-1^, respectively. A good indicator of the capacity and power of the glycolytic anaerobic source and body tolerance for lactic acid build up is blood lactic acid level (LA) after maximum exercise. This level was 11.31 mmol ·L^-1^ in judokas, which is 2.92 mmol ·L^-1^ lower than in the untrained subjects (14.23 mmol ·L^-1^). In both cases, these values were lower than in top world judokas [3] and similar to Polish athletes [13, 24, 40].

The experiments performed also demonstrated interaction between the group factor and HGS measurement conditions factor at all three time points. A statistically significant difference was found in judoists between HGSmax before warm-up and HGSmax after the WAnT test. The results were not consistent with previous studies, which demonstrated a decline in HGSmax after effort in a judo bout [26]. The longer and more specific exercise used in previous research procedures caused greater fatigue in the forearm muscles in judo athletes compared to our study. However, the present results were consistent with recent findings by Dias et al. [41], who demonstrated that judokas were not stronger than non-judokas in terms of HGSmax. The present results also showed an advantage of judokas in maintaining hand grip. This conclusion is partly consistent with findings obtained from the Dynamic Judogi Strength Endurance Test [19].

The measurements of the perceived force of ½HGSmax depended on interactions between the group factor and conditions of measurement. Before the warm-up (T1), athletes demonstrated higher force (more divergent from the calculated ½HGSmax value) compared to the untrained university students who performed this task more accurately at this stage of the experiment. A significant intergroup difference was observed for time point T1, pointing to worse hand grip force sensibility compared to the grip used at T1. Judo athletes, with judo considered as a "catching" combat sport, are likely to mobilize hands more before the exercise than the untrained controls.

The major finding of the present study in terms of balance is that the anaerobic procedure used to induce substantial fatigue in the human body significantly influences the deterioration of most of the stability indices analysed. There were particularly significant changes concerning sway path (SP) and mean velocity of displacement of the COP (MV) in both judokas and untrained subjects. Only mean amplitude (MA) and mean frequency of the amplitude of the COP (MF) turned out to be insensitive to muscular fatigue. In the experiment, the group of untrained subjects had worse stability indices compared to judokas, regardless of the conditions of performing the task (EO or EC). Changes in the conditions of the test through exclusion of visual control did not cause a significant deterioration in balance caused by increasing fatigue in any of the groups studied, and the Romberg indices did not differ significantly for any time point.

Our findings are consistent with the literature. Numerous studies have observed a significant deterioration in stability indices after intensive anaerobic exercise in both athletes in various sports and untrained subjects [35, 42–45]. Many of the studies have measured stability after the recovery period when maximal blood lactate levels were observed [42, 46]. Unequivocal impairment has been found in both the central and peripheral mechanisms of balance control [47].

In a more recent study [48], measurements of stability were performed immediately after the WAnT test. An important factor in such conditions that increases the indices is the internal disturbances generated through breathing mechanisms [49]. Intensified breathing activity might generate displacements of the centre of body mass and consequently displacements of the centre of gravity (COG). However, Golema [50] observed no effect on the stabilogram curve, even for forceful breathing and especially during the tests with eyes open. This effect can be observed in tests done with eyes closed. Compensatory movements were found in hip joints with the phase that was opposite to movements in knee joints, preventing loss of balance due to breathing movements. Therefore, the more important factor that impairs stability is changes that occur directly from the specific exercise and the following neuromuscular fatigue.

Some studies have reported a lack of significant correlations between the increase in blood lactate levels and deterioration of stability control [49, 51]. They found various types of compensatory mechanisms that limit instability under conditions of visual control. This can be attributed to the fact that training and the specific nature of a sport stimulates the mechanisms of improved postural control and tolerance for lactic acid build-up, especially in the case of team sports athletes. Our results are consistent with this and demonstrated a significantly higher effect of fatigue on untrained subjects, but they also unequivocally showed more substantial deterioration in the indices studied under conditions of visual control.

Analysis of the values of Romberg quotients (RQ) calculated for different time points reveals a low effect of fatigue on proprioceptors. The lack of significant differences in RQ between pre-test and post-test measurements and deterioration in most of the stability indices can be interpreted as a manifestation of the efferent rather the afferent neuromuscular fatigue, such as a delay in muscular response [42, 52,53].

The increase in instability after a warm-up is not consistent with reports by Brown and Bowyer of an increase in the effectiveness of exercise observed after moderate fatigue [54]. They emphasized improved efficiency of the mechanisms of flow of afferent and efferent impulses and function of muscle spindles in the stabilizing muscles of the talocrural joint, which contributes to individual improvement in balance. We found improvement in certain indices after the warm-up (MA; MV; SA; MF) in only the group of untrained subjects without visual control (EC). The lack of improvement in judoists might result from their high initial level of balance (or motivation before the expected effort). Intramuscular and intermuscular coordination are crucial for gaining power and strength. The balance training may include a strengthening effect and strength training balance effect in a closed kinematic chain [55].

The limitations of present study consist in that it is difficult to have access to elite-level athletes. Therefore, the number of such study participants was small. However, we did not want to extend the control group excessively in order not to disturb the proportions. We intend to continue our research in the future using Special Judo Fitness Test in bigger group of athletes but without fitness measurements before warm-up. The experiment will exclude untrained subjects since they are not familiar with judo throwing techniques. We expect to find large effects concerning disturbed balance but not for HGSmax or HGS sensitivity index.

## Conclusions

The present findings suggest specific training adaptations for judokas. These adaptations are especially manifested in body composition, efficiency of the anaerobic energy system, and postural balance. These specific abilities should be trained by judo athletes with consideration for time-motion and anaerobic demands of a contemporary judo fight. The fatigue induced by anaerobic exercise results in strong reduction in the effectiveness of the mechanisms of balance control. Smaller changes in trained vs. untrained study participants suggest the significant effect of the sport on minimization of unfavourable consequences of fatigue. It seems purposive to present guidelines to integrate balance drills with varied complexity into judokas' training programs. These drills should be performed not only at initial stages of training but also during the most intensive phases in order to stimulate the mechanisms of balance control under conditions of fatigue. Further research should be done into the effect of fatigue caused by anaerobic exercise on the dynamic balance.

## Supporting Information

S1 Data (XLSX)
